# Crystal structure of tin(II) perchlorate trihydrate

**DOI:** 10.1107/S1600536814024283

**Published:** 2014-11-12

**Authors:** Erik Hennings, Horst Schmidt, Martin Köhler, Wolfgang Voigt

**Affiliations:** a TU Bergakademie Freiberg, Institute of Inorganic Chemistry, Leipziger Strasse 29, D-09596 Freiberg, Germany

**Keywords:** crystal structure, low-temperature salt hydrates, perchlorate hydrates, tin(II) salts

## Abstract

The title compound, [Sn(H_2_O)_3_](ClO_4_)_2_, was synthesized by the redox reaction of copper(II) perchlorate hexa­hydrate and metallic tin in perchloric acid. Both the trigonal–pyramidal [Sn(H_2_O)_3_]^2+^ cations and tetra­hedral perchlorate anions lie on crystallographic threefold axes. In the crystal, the cations are linked to the anions by O—H⋯O hydrogen bonds, generating (001) sheets.

## Abstract 2

The title compound was synthesized by the redox reaction of copper(II) perchlorate hexa­hydrate and metallic tin in perchloric acid. Both the pyramidal [Sn(H_2_O)_3_]^2+^ cations and tetra­hedral perchlorate anions lie on crystallographic threefold axes.

## Chemical context   

The synthesis and powder diffraction data for tin(II) perchlorate trihydrate were described by Davies & Donaldson (1968 ▶) and Schiefelbein & Daugherty (1970 ▶). With our crystal structure determination, the data of Davies & Donaldson (1968 ▶) are confirmed. The inter­est in the system tin(II)–perchloric acid-water arose from the redetermination of the redox-potential Sn^2+^/Sn^4+^ in perchloric acid by Gajda *et al.* (2009 ▶). There is no solid–liquid diagram for this binary salt–water system known in the literature.

## Structural commentary   

**Figure 1:**
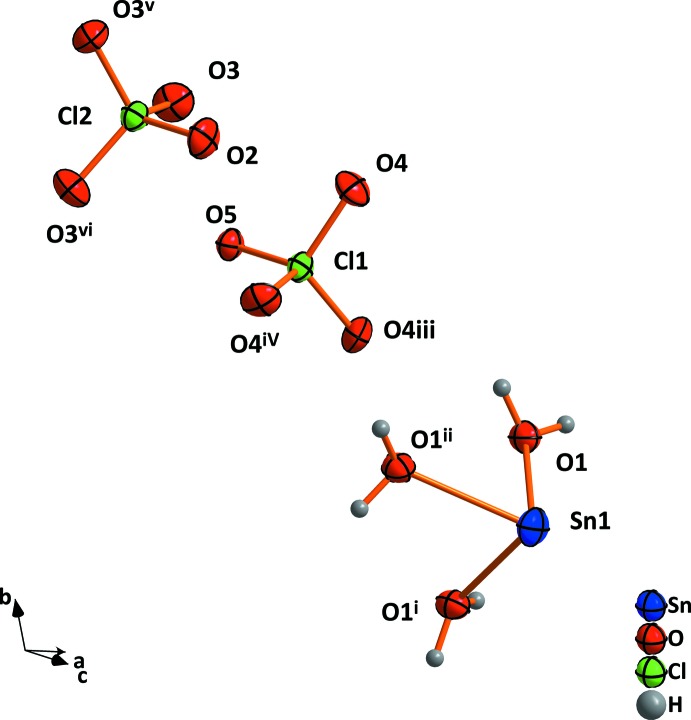
The component ions in tin(II) perchlorate trihydrate with displacement ellipsoids drawn at the 50% probability level. [Symmetry codes: (i) −*x* + *y*, −*x*, *z*; (ii) −*y*, *x* − *y*, *z*; (iii) 1 − *x* + *y*, 1 − *x*, *z*; (iv) 1 − *y*, *x* − *y*, *z*; (v) 1 − y, 1 + *x* − *y*, *z*; (vi) −*x* + *y*, 1 − *x*, *z*.]

The tin atom lies on a crystallographic threefold rotation axis and is coordinated by three water mol­ecules as a trigonal pyramid (Fig. 1 ▶, Table 1 ▶). The perchlorate tetra­hedra are located in the gaps between the SnO_3_ pyramids on their own threefold axes. A similar arrangement of the perchlorate tetra­hedra can be observed in the crystal structure of Ba(ClO_4_)_2_·3H_2_O (Gallucci & Gerkin, 1988 ▶). The difference between the two structures is that the barium atom is sixfold coordinated by oxygen water mol­ecules. All of them are shared between two barium atoms, so that an average of three are bonded to one Ba atom.

## Supra­molecular features   

**Figure 2:**
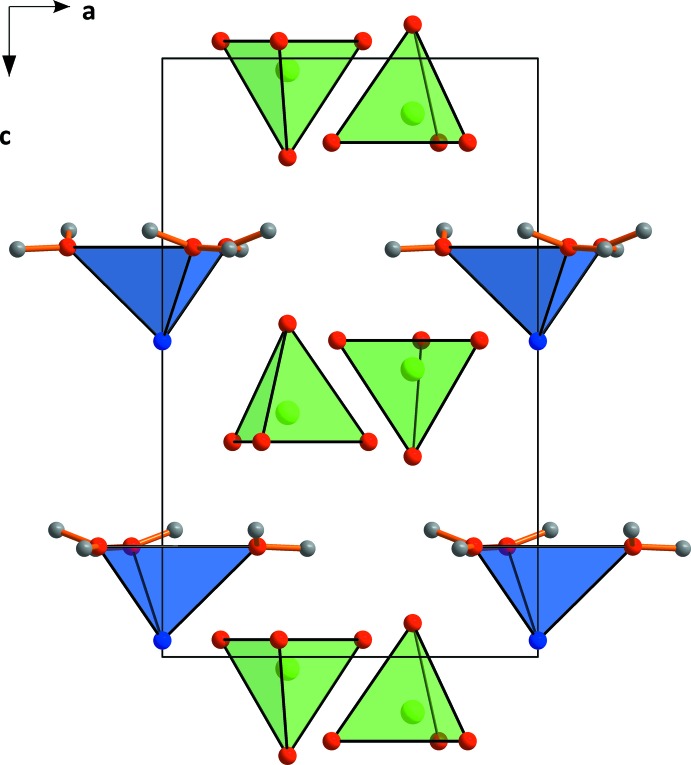
The unit-cell packing in tin(II) perchlorate trihydrate with the ions shown in polyhedral representation.

The different coordination of Sn^2+^ in comparison with Ba^2+^ is caused by the lone-pair effect. It requires more space, so the distance to the next oxygen atoms is larger than in the barium salt structure. The perchlorate tetra­hedra are connected by O—H⋯O hydrogen bonds (Table 2 ▶) with the water mol­ecules coordinated at the tin atoms (Figs. 2 ▶ and 3 ▶), forming sheets parallel to (001).

## Database survey   

For properties, thermal behavior and powder diffraction data for tin(II) perchlorate trihydrate, see: Schiefelbein & Daugherty (1970 ▶) and Davies & Donaldson (1968 ▶). For crystal structure determinations of other divalent perchlorate trihydrates, see: Gallucci & Gerkin (1988 ▶) for the barium salt and Hennings *et al.* (2014 ▶) for the strontium salt.

## Synthesis and crystallization   

Sn(ClO_4_)_2_·3H_2_O was prepared by reaction of copper(II) perchlorate hexa­hydrate (15 g, Alfa Aesar, reagent grade) and elemental tin (12.04 g, VEB Feinchemikalien) in perchloric acid (50 ml, 60%, Merck, pA). After stirring the solution for 2 h the precipitated copper was filtered off and the solution was transferred into a freezer at 253 K for crystallization. All crystals are stable in the saturated aqueous solution over a period of at least four weeks.

The sample was stored in a freezer or a cryostat at low temperatures. The crystals were separated and embedded in perfluorinated ether for X-ray analysis.

## Refinement   

Crystal data, data collection and structure refinement details are summarized in Table 3 ▶. The H atoms were placed in the positions indicated by difference Fourier maps. No further constraints were applied.

## Supplementary Material

Crystal structure: contains datablock(s) I. DOI: 10.1107/S1600536814024283/hb7297sup1.cif

Structure factors: contains datablock(s) I. DOI: 10.1107/S1600536814024283/hb7297Isup2.hkl

CCDC reference: 1032662

Additional supporting information:  crystallographic information; 3D view; checkCIF report

## Figures and Tables

**Figure 3:**
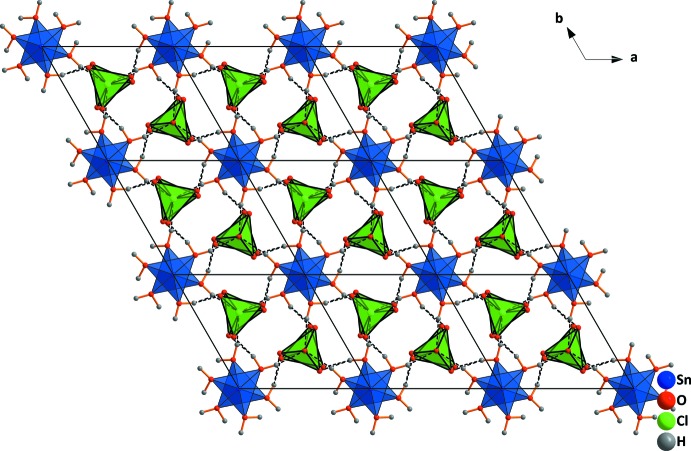
Larger view of the crystal structure of tin(II) perchlorate trihydrate viewed down [001]. Dashed lines indicate hydrogen bonds.

**Table 1:** Selected geometric parameters (, )

Sn1O2	2.201(7)	Cl2O1	1.424(12)
Cl1O4	1.430(4)	Cl2O3	1.426(5)
Cl1O5	1.449(10)		
			
O2^i^Sn1O2	76.9(3)		

Symmetry code: (i) 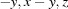.

**Table 2:** Hydrogen-bond geometry (, )

*D*H*A*	*D*H	H*A*	*D* *A*	*D*H*A*
O2H2O4^ii^	0.94(7)	1.95(8)	2.823(8)	152(7)
O2H2O3^iii^	0.94(7)	2.46(8)	2.926(8)	110(6)

Symmetry codes: (ii) 
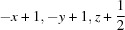
; (iii) 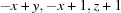.

**Table 3:** Experimental details

Crystal data	
Chemical formula	[Sn(H_2_O)_3_](ClO_4_)_2_
*M*_r_	371.44
Crystal system, space group	Hexagonal, *P*6_3_
Temperature (K)	180
*a*, *c* ()	7.0701(10), 9.7631(15)
*V* (^3^)	422.64(16)
*Z*	2
Radiation type	Mo *K*
(mm^1^)	3.70
Crystal size (mm)	0.70 0.52 0.22

Data collection	
Diffractometer	STOE IPDS 2
Absorption correction	Integration (Coppens, 1970 ▶)
*T*_min_, *T*_max_	0.116, 0.441
No. of measured, independent and observed [*I* > 2(*I*)] reflections	792, 788, 742
*R*_int_	0.152
(sin /)_max_ (^1^)	0.689

Refinement	
*R*[*F* ^2^ > 2(*F* ^2^)], *wR*(*F* ^2^), *S*	0.036, 0.093, 1.08
No. of reflections	792
No. of parameters	52
No. of restraints	1
H-atom treatment	All H-atom parameters refined
_max_, _min_ (e ^3^)	0.85, 0.90
Absolute structure	Classical Flack (1983 ▶) method preferred over Parsons Flack (2004 ▶) because s.u. lower
Absolute structure parameter	0.04(14)

Computer programs: *X-AREA* and *X-RED* (Stoe Cie, 2009 ▶), *SHELXS97* and *SHELXL2012* (Sheldrick, 2008 ▶), *DIAMOND* (Brandenburg, 2006 ▶) and *publCIF* (Westrip, 2010 ▶).

## supplementary crystallographic information

### Crystal data

**Table:**

[Sn(H_2_O)_3_](ClO_4_)_2_	*D*_x_ = 2.919 Mg m^−^^3^
*M**_r_* = 371.44	Mo *K*α radiation, λ = 0.71073 Å
Hexagonal, *P*6_3_	Cell parameters from 14633 reflections
*a* = 7.0701 (10) Å	θ = 2.1–29.6°
*c* = 9.7631 (15) Å	µ = 3.70 mm^−^^1^
*V* = 422.64 (16) Å^3^	*T* = 180 K
*Z* = 2	Prism, colourless
*F*(000) = 355.8	0.70 × 0.52 × 0.22 mm

### Data collection

**Table:**

STOE IPDS 2 diffractometer	788 independent reflections
Radiation source: fine-focus sealed tube	742 reflections with *I* > 2σ(*I*)
Detector resolution: 6.67 pixels mm^-1^	*R*_int_ = 0.152
rotation method scans	θ_max_ = 29.3°, θ_min_ = 3.3°
Absorption correction: integration (Coppens, 1970)	*h* = −7→9
*T*_min_ = 0.116, *T*_max_ = 0.441	*k* = 0→9
792 measured reflections	*l* = −13→13

### Refinement

**Table:**

Refinement on *F*^2^	All H-atom parameters refined
Least-squares matrix: full	*w* = 1/[σ^2^(*F*_o_^2^) + (0.0771*P*)^2^] where *P* = (*F*_o_^2^ + 2*F*_c_^2^)/3
*R*[*F*^2^ > 2σ(*F*^2^)] = 0.036	(Δ/σ)_max_ < 0.001
*wR*(*F*^2^) = 0.093	Δρ_max_ = 0.85 e Å^−^^3^
*S* = 1.08	Δρ_min_ = −0.90 e Å^−^^3^
792 reflections	Extinction correction: *SHELXL*, Fc^*^=kFc[1+0.001xFc^2^λ^3^/sin(2θ)]^-1/4^
52 parameters	Extinction coefficient: 0.62 (5)
1 restraint	Absolute structure: Classical Flack (1983) method preferred over Parsons & Flack (2004) because s.u. lower.
Hydrogen site location: difference Fourier map	Absolute structure parameter: −0.04 (14)

### Special details

**Table:**

Geometry. All e.s.d.'s (except the e.s.d. in the dihedral angle between two l.s. planes) are estimated using the full covariance matrix. The cell e.s.d.'s are taken into account individually in the estimation of e.s.d.'s in distances, angles and torsion angles; correlations between e.s.d.'s in cell parameters are only used when they are defined by crystal symmetry. An approximate (isotropic) treatment of cell e.s.d.'s is used for estimating e.s.d.'s involving l.s. planes.

### Fractional atomic coordinates and isotropic or equivalent isotropic displacement parameters (Å^2^)

**Table:**

	*x*	*y*	*z*	*U*_iso_*/*U*_eq_	
Sn1	0.0000	0.0000	0.9724 (6)	0.0280 (4)	
O2	0.2529 (5)	0.1712 (6)	0.8156 (8)	0.0239 (8)	
O3	0.5340 (6)	0.6895 (7)	−0.0288 (9)	0.0311 (10)	
Cl1	0.6667	0.3333	0.0921 (2)	0.0182 (5)	
Cl2	0.3333	0.6667	0.0195 (3)	0.0197 (5)	
O1	0.3333	0.6667	0.1653 (12)	0.0295 (18)	
O4	0.8132 (6)	0.5490 (7)	0.1408 (7)	0.0280 (10)	
O5	0.6667	0.3333	−0.0564 (10)	0.0204 (16)	
H1	0.386 (19)	0.209 (16)	0.83 (2)	0.06 (3)*	
H2	0.246 (11)	0.293 (10)	0.783 (8)	0.017 (17)*	

### Atomic displacement parameters (Å^2^)

**Table:**

	*U*^11^	*U*^22^	*U*^33^	*U*^12^	*U*^13^	*U*^23^
Sn1	0.0337 (4)	0.0337 (4)	0.0164 (5)	0.0169 (2)	0.000	0.000
O2	0.0197 (15)	0.0232 (14)	0.030 (2)	0.0113 (12)	−0.0002 (19)	0.001 (2)
O3	0.0264 (16)	0.0359 (18)	0.034 (2)	0.0178 (15)	0.009 (3)	0.005 (3)
Cl1	0.0200 (7)	0.0200 (7)	0.0146 (12)	0.0100 (3)	0.000	0.000
Cl2	0.0202 (7)	0.0202 (7)	0.0187 (13)	0.0101 (3)	0.000	0.000
O1	0.038 (3)	0.038 (3)	0.013 (5)	0.0190 (15)	0.000	0.000
O4	0.0301 (18)	0.0236 (17)	0.028 (2)	0.0118 (14)	−0.003 (2)	−0.010 (2)
O5	0.023 (2)	0.023 (2)	0.016 (4)	0.0114 (11)	0.000	0.000

### Geometric parameters (Å, º)

**Table:**

Sn1—O2^i^	2.201 (7)	Cl1—O5	1.449 (10)
Sn1—O2^ii^	2.201 (7)	Cl2—O1	1.424 (12)
Sn1—O2	2.201 (7)	Cl2—O3^v^	1.426 (5)
Cl1—O4	1.430 (4)	Cl2—O3^vi^	1.426 (5)
Cl1—O4^iii^	1.430 (4)	Cl2—O3	1.426 (5)
Cl1—O4^iv^	1.430 (4)		
			
O2^i^—Sn1—O2^ii^	76.9 (3)	O4^iv^—Cl1—O5	109.4 (3)
O2^i^—Sn1—O2	76.9 (3)	O1—Cl2—O3^v^	109.3 (4)
O2^ii^—Sn1—O2	76.9 (3)	O1—Cl2—O3^vi^	109.3 (4)
O4—Cl1—O4^iii^	109.5 (3)	O3^v^—Cl2—O3^vi^	109.6 (4)
O4—Cl1—O4^iv^	109.5 (3)	O1—Cl2—O3	109.3 (4)
O4^iii^—Cl1—O4^iv^	109.5 (3)	O3^v^—Cl2—O3	109.6 (4)
O4—Cl1—O5	109.4 (3)	O3^vi^—Cl2—O3	109.6 (4)
O4^iii^—Cl1—O5	109.4 (3)		

Symmetry codes: (i) −*x*+*y*, −*x*, *z*; (ii) −*y*, *x*−*y*, *z*; (iii) −*x*+*y*+1, −*x*+1, *z*; (iv) −*y*+1, *x*−*y*, *z*; (v) −*y*+1, *x*−*y*+1, *z*; (vi) −*x*+*y*, −*x*+1, *z*.

### Hydrogen-bond geometry (Å, º)

**Table:**

*D*—H···*A*	*D*—H	H···*A*	*D*···*A*	*D*—H···*A*
O2—H2···O4^vii^	0.94 (7)	1.95 (8)	2.823 (8)	152 (7)
O2—H2···O3^viii^	0.94 (7)	2.46 (8)	2.926 (8)	110 (6)

Symmetry codes: (vii) −*x*+1, −*y*+1, *z*+1/2; (viii) −*x*+*y*, −*x*+1, *z*+1.
